# Emerging Perspectives on Prime Editor Delivery to the Brain

**DOI:** 10.3390/ph17060763

**Published:** 2024-06-11

**Authors:** Eli BenDavid, Sina Ramezanian, Yaoyao Lu, Joël Rousseau, Avi Schroeder, Marc Lavertu, Jacques P. Tremblay

**Affiliations:** 1 Laboratory of Biomaterials and Tissue Engineering, Department of Chemical Engineering, Institute of Biomedical Engineering, Polytechnique Montréal, Montréal, QC H3C 3A7, Canada; marc.lavertu@polymtl.ca; 2 Division of Human Genetics, Centre de Recherche du CHU de Québec—Université Laval, Québec, QC G1V 4G2, Canada; 3 Laboratory of Molecular Genetics and Gene Therapy, Department of Molecular Medicine, Faculty of Medicine, Laval University, Québec, QC G1V 0A6, Canada; 4 Laboratory of Nanopharmacology and Pharmaceutical Nanoscience, Faculty of Pharmacy, Laval University, Québec, QC G1V 4G2, Canada; 5 Rappaport Faculty of Medicine, Technion—Israel Institute of Technology, Haifa 3525433, Israel; 6 Laboratory for Targeted Drug Delivery and Personalized Medicine Technologies, Department of Chemical Engineering, Technion—Israel Institute of Technology, Haifa 3200003, Israel; avids@technion.ac.il

**Keywords:** neurology, gene therapy, CRISPR/Cas9, prime editing, genetic engineering, drug delivery system, nanomaterial, targeted drug delivery, personalized nanomedicine

## Abstract

Prime editing shows potential as a precision genome editing technology, as well as the potential to advance the development of next-generation nanomedicine for addressing neurological disorders. However, turning in prime editors (PEs), which are macromolecular complexes composed of CRISPR/Cas9 nickase fused with a reverse transcriptase and a prime editing guide RNA (pegRNA), to the brain remains a considerable challenge due to physiological obstacles, including the blood–brain barrier (BBB). This review article offers an up-to-date overview and perspective on the latest technologies and strategies for the precision delivery of PEs to the brain and passage through blood barriers. Furthermore, it delves into the scientific significance and possible therapeutic applications of prime editing in conditions related to neurological diseases. It is targeted at clinicians and clinical researchers working on advancing precision nanomedicine for neuropathologies.

## 1. Introduction

Gene therapy, which was initially proposed by Friedmann and Roblin to address genetic disorders, holds promising potential as a method to address gene disorders [1]. Unlike conventional medications, gene therapy has the potential to address the etiology of diseases by introducing normal genes, correcting a mutated gene, or inhibiting the expression of faulty genes to prevent or alleviate symptoms [2]. The CRISPR-/Cas9-derived prime editor (PE), which is a technology that was referred to as the “search-and-replace” technique by its founders [3], brings hope for deploying other precision gene editing technologies for many pathologies. Among the CRISPR/Cas9 genome editing technologies, prime editing provides exceptional flexibility by allowing for the precision substitution of a target DNA sequence with nearly any other specified sequence containing up to several hundred inserted, deleted, or substituted base pairs [4,5]. Prime editing technology enables all 12 potential base-to-base conversions, as well as insertions and deletions, without requiring double-strand DNA breaks (DBSs) or donor DNA [3,6,7]. Prime editing is distinguished from traditional CRISPR-Cas9 frameworks by its minimized collateral genomic impacts [8,9].

This innovation holds promise in revolutionizing gene therapy for neurological conditions by enabling the precise correction of harmful mutations and targeted manipulation of gene activity [9]. Neurological diseases, including Parkinson’s disease (PD), Alzheimer’s disease (AD), Huntington‘s disease (HD), autism spectrum disorder (ASD), malignant neoplasm (i.e., glioblastomas), and strokes are complex nervous system diseases influenced by a variety of factors, such as age, genetic and environmental factors, oxidative stress, mitochondrial dysfunction, and neuro-inflammation, which are all contribute to the development of their pathologies [5,6,7]. However, many of those pathologies are genetically predisposed; some are brought on by a single nucleotide variant, while others have been shown to have complicated modes of inheritance. A meta-analysis has shown that many of the single-nucleotide polymorphisms associated with longevity and cognitive performance are associated with the development of neurodegenerative diseases [8,9]. For instance, it has been reported that the DNA mutations involved in the gene that codes for amyloid-beta precursor protein (APP) are responsible for some forms of AD. Moreover, scientists have demonstrated that the APP mutations mapping to exons 16 and 17 contribute to plaque accumulation and cause familial Alzheimer’s disease (FAD) [10,11].

Other neurodegenerative pathologies, such as HD [12], Friedreich ataxia, and spinocerebellar atrophy [13], contain a dynamic mutation, leading to the development of DNA sequence repeats. These DNA repeats either affect the translation of the corresponding gene and subsequently decrease the protein’s expression or impair the protein’s normal function.

Evidence indicates that neurodevelopmental disorders in childhood, such as intellectual disability, ASD, and attention-deficit/hyperactivity disorder (ADHD), share genetic risk genes. These common genetic variables are also associated with psychiatric illnesses, including schizophrenia [12]. Individuals with schizophrenia exhibit a notable increase in copy number variants linked to intellectual disability [14,15]. This suggests that certain genetic variations connected with intellectual disability may also play a role in the development of schizophrenia, although to a lesser extent [14]. Moreover, some forms of ASD can be attributed to mutations in a single gene, which offers potential targets for gene therapy interventions by prime editing (Table 1 and Table 2).

Although prime editing technology is now in its preliminary phases, its prospective clinical applications are extensive and have the potential to transform the treatment of several neurological pathologies, as indicated earlier. The first clinical trial using this technology for gene therapy is expected to be carried out by its co-founders in early 2024 [16].

**Table 1:** Monogenic syndromic autism spectrum disorder.

Syndrome	Gene	Cause	Frequency	Refs.
Angelman	*UBE3A*	SV *, Loss of allele	Between 1/12,000 and 1/20,000	[17,18]
Prader–Willi	*Magel2*	Loss	1/15	[19,20]
Fragile X	*FMR1*	Repeat expansion	Between 1/7000 and 1/11,000	[21,22]
Undetermined	*SHANK2*	Missense, SV missense	-	[18,23]
Phelan–McDermid	*SHANK3*	Deletion	Between 2 and 10 of every 1 million	[24,25]
Rett Syndrome	*MECP2*	Mutation, Indels	Between 1/10,000 and 1/15,000	[26,27]
Dias–Logan syndrome	*BCL11A*	Deletion	-	[25,28]
Undetermined	*NLGN3*	SV, duplication	-	[29,30]
Undetermined	*NLGN4X*	Missense, truncating	-	[18,25,29]
Undetermined	*NLGN4Y*	Missense, truncating	-	[31,32]
15q11–q13	*GABA_A_* receptor genes cluster, *UBE3A, CYFIP1*	Duplication	-	[25,33]

* Structural varian.

**Table 2:** Potential neurodegenerative disease genes of interest for prime editing.

Pathologies	Potential Gene to Target	Refs.
Alzheimer’s Disease	*APP*, *PSEN1*, *PSEN2*, *APOE ε4*	[34,35,36,37,38,39]
Parkinson’s Disease	*SNCA*, *LRRK2*, *VPS35*, *PRKN*, *PINK1*, *DJ1*	[40,41,42,43,44,45,46,47,48]
Amyotrophic Lateral Sclerosis	*ANXA11*, *ARPP21*, *CAV1*, *C21ORF2*, *CCNF*, *DNAJC7*, *GLT8D1*, *KIF5A*, *NEK1*, *SPTLC1*, *TIA1*	[49,50,51,52,53,54,55,56,57,58]
Tay Sachs Disease	*HEXA*	[59,60,61]
Huntington’s Disease	*HTT*	[62,63,64]
Duchenne Muscular Dystrophy Spinal Muscular Atrophy	*DMD* *SMN1*	[65,66,67,68]
Friedreich’s Ataxia	*FXN*	[69,70]

## 2. Prime Editing System

**Figure 1:**
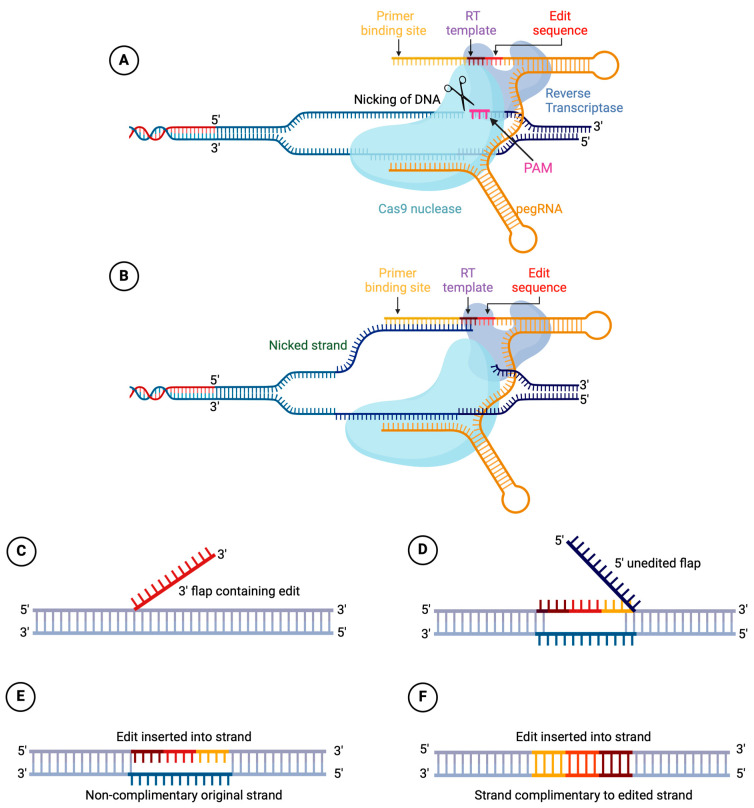
Prime editing system construct and editing mechanism. (**A**) Spacer sequence of pegRNA guides the complex to the target site and the complex binds to the DNA. Cas9 identifies a PAM and nicks 3 nucleotides upstream of the PAM. (**B**) PBS binds to nicked genomic DNA, and RT uses RTT as a template to copy the edit into a 3′DNA flap. The edited 3′-flap (**C**) competes with the original 5′-flap (**D**) for binding to the target DNA. If a 3′-flap occurs, the desired edit will be kept (**E**); however, editing will be lost if the 5′-flap happens (**F**) [3,80]. Created with BioRender.com, accessed on 7 May 2024 (Agreement Number WW26VSF5P1). Adopted with permission from Dr. Jacques P. Tremblay, Successful Correction by Prime Editing of a Mutation in the RYR1 Gene Responsible for a Myopathy; published by MDPI Cells, 2024.

Prime editing, as depicted in Figure 1, is composed of a catalytically impaired SpCas9 nickase (SpCas9n (H840A)) linked to an engineered reverse transcriptase (RT), along with a prime editing guide RNA (pegRNA) [3,4]. pegRNA comprises the following three crucial subsequences: a guide sequence (spacer), a primer binding site (PBS), and a reverse transcription template (RTT) [71]. The spacer guides the pegRNA to the specific location in the genome called the protospacer, in which Cas9 recognizes a protospacer-adjustment motive (PAM) and triggers a single-strand break three nucleotides upstream. Next, the PBS hybridizes to its complementary sequence, the nicked 3′ end of the target DNA, serving as a primer for RT. Subsequently, the RT utilizes the RTT as a template to introduce the target edit onto the nontarget strand. At this stage, one of the DNA strands contains a duplicated section. An edited 3′-flap can then ligate onto the target strand, displacing the unedited 5′-flap. If a 3′-flap occurs, the correction will be retained. However, if a 5′-flap occurs, the edit will be lost [3]. To date, seven generations of prime editors (PE) have been developed. PE1 has lower efficiency compared to others because it includes a wild-type variant of RT; PE2 uses an engineered variant of the M-MLV RT, which increases editing efficiency; PE3 uses an additional nicking sgRNA (nsgRNA) to introduce a nick in the non-edited strand, leading to further improved editing efficiencies (PE2 + nsgRNA) [3]. PE4 and PE5 involve the co-expression of MLH1dn, an engineered variant of a mismatch repair (MMR)-inhibiting protein, with PE2 and PE3, respectively [72]. PE6 is a shorter and more efficient PE, which was generated using phage-assisted protein evolution and engineering [73]. PE7 is a PE protein fused to the RNA-binding, N-terminal domain of La. La is a small RNA-binding protein that improves prime editing efficiency. La promotes the stability and integrity of pegRNAs via interacting with the 3′ ends of pegRNAs [74].

These distinctive attributes render prime editing a formidable candidate in the pursuit of gene-specific therapeutic strategies for neurological pathologies, offering avenues for the corrective amendment of pathogenic mutations and the reinstatement of normal gene operations.

**Figure 2:**
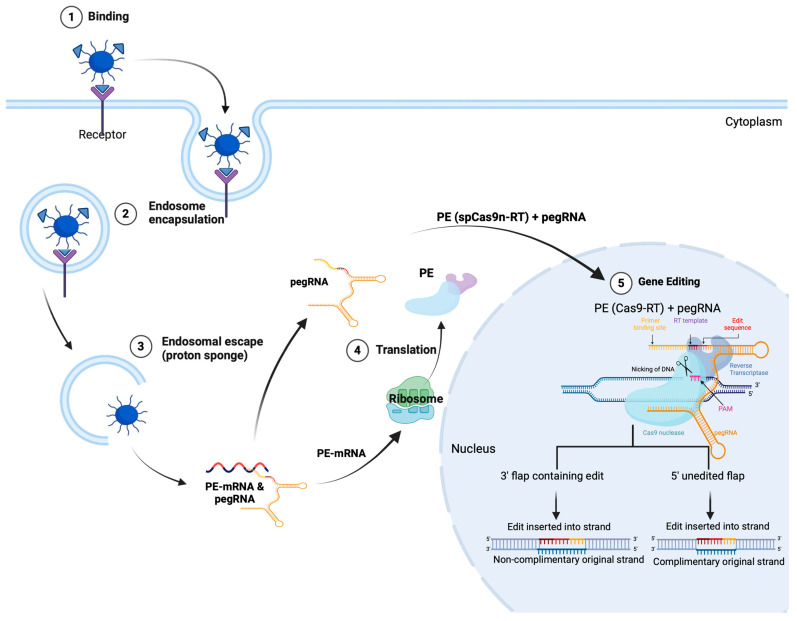
Illustration of cellular uptake and action mechanism of encapsulated PE (mRNA form) by cationic nanoparticles. Created with BioRender.com, accessed on 7 May 2024 (Agreement Number GS26VSESP3).

Given the proven efficacy of the mRNA-based vaccines of BioNTech/Pfizer and Moderna [75], along with their approval by health and drug authorities like Health Canada and the United States Food and Drug Administration [76,77], coupled with promising results, suggest that mRNA-based therapeutics will be a logical approach for treating and preventing various diseases. This could potentially translate into the preferred form of the prime editing system (Figure 1 and Figure 2) [78]. mRNA, a linear polymer, is an anionic polyelectrolyte composed of nucleotide units linked by covalent bonds. These polymers have a phosphoryl end, called the 5′ end, and a hydroxyl end, or 3′ end. Each mRNA nucleotide comprises a ribose linked to a phosphate group and a nitrogen base. Phosphate groups are present at regular intervals throughout the mRNA chain. Since the pKa of the phosphate group is close to 0, this functional group maintains a negative charge in a physiological environment [79]. The anionic nature of nucleic acids like mRNA can be exploited to develop nanoparticles with cationic materials.

There have been few studies using the prime editing system for neurodegenerative diseases. For instance, Tremblay et al. utilized PE to introduce the prophylactic A673T mutation, also known as the Icelandic Protective mutation, in the APP gene to prevent the β-secretase cleavage [10,81]. In their in vitro study on HEK293T cell lines, the researchers achieved the introduction of the A673T mutation in the cells at a frequency of 6% with PE2, 9.9% with PE3, and 25% by reengineering the PAM sequence and using PE3. Additionally, after conducting ten repetitions of the last prime editing treatment, they achieved an editing efficiency of 65% [10].

Mbakam et al. [77] conducted research on therapeutic gene therapy for Duchenne muscular dystrophy (DMD) and demonstrated the efficacy of prime editing technology in generating precise point mutations in the *DMD* gene. They validated previous discoveries and underscored the importance of further experiments prior to clinical trials. The PE2 and PE3 yielded up to 11% and 21% of targeted mutations in the *DMD* gene within HEK293T cells, respectively. Additionally, they found that introducing an extra mutation to the PAM sequence enhanced the PE3 outcome to 38% following a single treatment. This correction of myoblasts resulted in dystrophin expression detected through Western blot analysis.

## 3. Challenges of Delivering Prime Editors to the Brain

**Figure 3:**
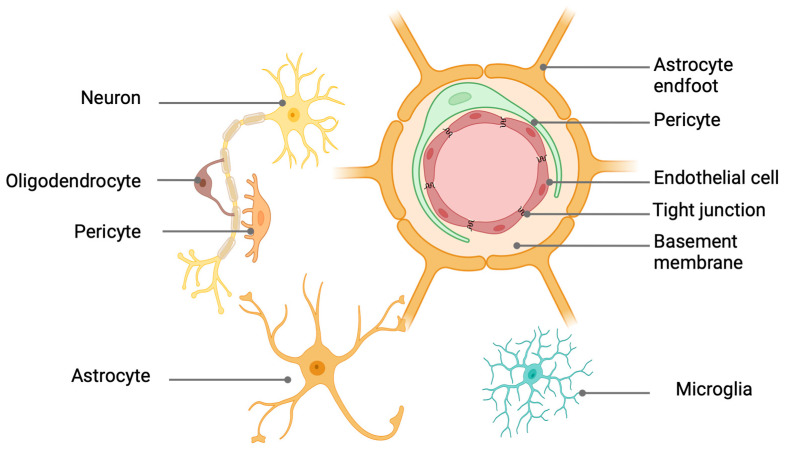
Neurovascular unit. The NVU consists of various types of cells closely connected and working together to preserve an optimal neuronal environment. Cerebral endothelial cells, which make up the BBB, form tight junctions that control the paracellular pathway. Pericytes partially surround these endothelial cells and share a basal lamina with them. Astrocytes cover the microvessel wall, playing crucial roles in barrier development and maintenance while also establishing connections with neurons. Microglia, resident immune cells within the CNS, have highly mobile cellular processes that can come into contact with astrocyte basal lamina [86]. Created with BioRender.com, accessed on 7 May 2024 (Agreement Number EW26VQL1FE).

Challenges emerge in delivering nanomedicine for certain neuropathologies that necessitate targeting the brain, such as AD, PD, and ASD. The brain is protected by complex and finely regulated biological barriers that prevent harmful substances from entering the brain. These barriers include the blood–brain barrier (BBB), blood–cerebrospinal fluid barrier (BCSFB), and arachnoid barrier [82]. These three layers act to restrict and control the movement of molecules at the interfaces between blood and neural tissue or its fluid spaces protecting the brain from potentially harmful substances [83]. The BBB imposes the most rigorous regulation on the brain’s microenvironment, making it a more substantial obstacle compared to other barriers [84]. The BBB forms the core of the neurovascular unit (NUV) and consists of brain microvessel endothelial cells, extracellular matrix, pericytes, and astrocytes. The NUV is the system responsible for the selective permeability of BBB (Figure 3) [82,85].

Understanding the full extent of the impact of the BBB on drug delivery to the CNS, especially to the brain, has been a long-standing challenge that persists today. However, it is established that the drug efficacy, when administered systematically (i.e., intravenously, i.v.), is reduced due to the inability of drugs to traverse the BBB. According to various studies, approximately 98% of neurotherapeutic agents cannot cross these barriers, making them a significant obstacle in developing and delivering effective treatments for CNS diseases [87], mainly due to the blood barrier’s selective permeability and enzymatic properties [88]. The BBB blocks nearly 100% of macromolecules [87].

Due to blood barriers’ selective permeability, only drugs with particular characteristics can successfully bypass them without eliciting elimination or immune responses, complicating the ability to control drug dose–effect relationships. The clinical ineffectiveness of many pharmacological interventions targeting the CNS is frequently due to the drugs’ failure to attain sufficient concentrations within the CNS [89,90,91,92]. Consequently, optimizing the controlled release and dose–response relationship is crucial for the successful delivery of PE to brain tissues, as well as the overall effectiveness of treatment.

Research has also shown that aging, cerebrovascular accident (CVA), carcinogenetic neoplasms, infections, injuries, ischemic events, neuroinflammation, neurodegenerative diseases (i.e., AD), and hydrocephalus may compromise these protective barrier structures, permeability, and functionality and potentially increase the BBB’s permeability [93,94,95]. Moreover, in vitro studies on SARS-COVID-19 SARS-CoV-2 pathogens have indicated that the pathogens’ S1 and S2 spike proteins trigger a pro-inflammatory reaction in brain endothelial cells, potentially leading to alterations in BBB functionality [96,97,98,99,100]. Others have reported that SARS-CoV-2 can penetrate the BBB through a transcellular route, often resulting in the disruption of the basement membrane without causing significant changes to tight junctions [101].

Altered function and uncertainty in the BBB’s response to a disease have presented new challenges for delivering PE to the brain, especially following the recent pandemic. The clinical bedside significance of variability in permeability of BBB in the broader context of drug design becomes particularly relevant. The therapeutic window and therapeutic index can vary from one patient to another. The critical question then arises on how a clinician can calibrate the prescribed therapeutic dosage for patient(s) that the dosage and its strength either results in potential side effects or leads to therapeutic inefficacy, which is both clinically and ethically devastating. Consequently, we urgently need to reassess our approaches for transporting PE to the brain given the BBB long-standing challenge.

## 4. Drug Administration Routes

A medication’s administration route directly influences the drug’s bioavailability, which dictates its debut and the duration of the intended pharmacological action [102]. The choice of route of administration may be impacted by various circumstances, such as the medical history and status of the patient and their choice of therapeutic route, age, demographics, etc. [103]. In general, there are two administration routes for any given drug, as follows: (i) local route and (ii) systemic route. Local administration is simply administering a drug to the intended target site. Some examples of this administration route are skin topical, intranasal, ocular drops, etc. [104,105,106]. Systemic routes are delivered via enteral (i.e., oral, sublingual, rectal) or parenteral (i.e., inhalational, transdermal, injections) [107] methods. The parenteral administration route via injections has gained much attention because it is practical and non-invasive. Intravenous (i.v.), intra-arterial (i.a.), intrathecal (i.t.), intracelebrovascular (i.c.v.), etc. are the most tested methods for CNS drug administration [108,109]; however the i.t. and i.c.v. routes are considered invasive.

Moreover, in systemic absorptions, sometimes multiple routes are used to generate an effective pharmacological effect [110,111,112]. The research trend into developing drugs for the CNS is now focused on either improving systemic medication delivery to the brain or finding ways to overcome or disrupt the BBB. Even though i.v. has been the standard administration route, there are other strategies besides i.v., including surgical (i.e., convection-enhanced delivery, intra-arterial infusion, osmotic BBB disruption, targeted ultrasound BBB disruption) and pharmacological (i.e., bradykinin analogs, receptor-mediated transcytosis, P-glycoprotein (PgP) inhibitors) approaches, that allow for the therapeutic passage through blood barriers [85,113,114,115,116,117].

A clinical trial testing neurotrophin gene therapy for AD showed no positive outcome when an adeno-associated virus (AAV2) carrying nerve growth factor was injected directly into the brain [118]. Strategies such as the i.c.v. injection are invasive and in the experimental phase or reaching preclinical and early clinical phases, and therefore, their clinical implications and risk–benefit for gene therapy are yet to be fully assessed. Moreover, the lack of substantial data extends to their efficacy for gene therapy, mainly to transport a macromolecule such as a PE system (i.e., SpCas9n-RT (mRNA form) and epegRNA). The delivery of PE will pose similar challenges associated with the BBB, as well as other complications, including rapid clearance in the blood, poor cellular uptake, large size of PE molecule (i.e., 6–7 kb), limited specificity, and off-target effects [119].

In response to these obstacles and ambiguities, innovative approaches and methodologies are under exploration, including the use of tailored viral vectors, nanoparticles, and leveraging receptor-mediated transcytosis [120]. These emerging strategies endeavor to improve the efficiency of PE transference into cerebral zones while aiming to curtail unintended effects and immune rejections.

## 5. Drug Delivery Systems

**Figure 4:**
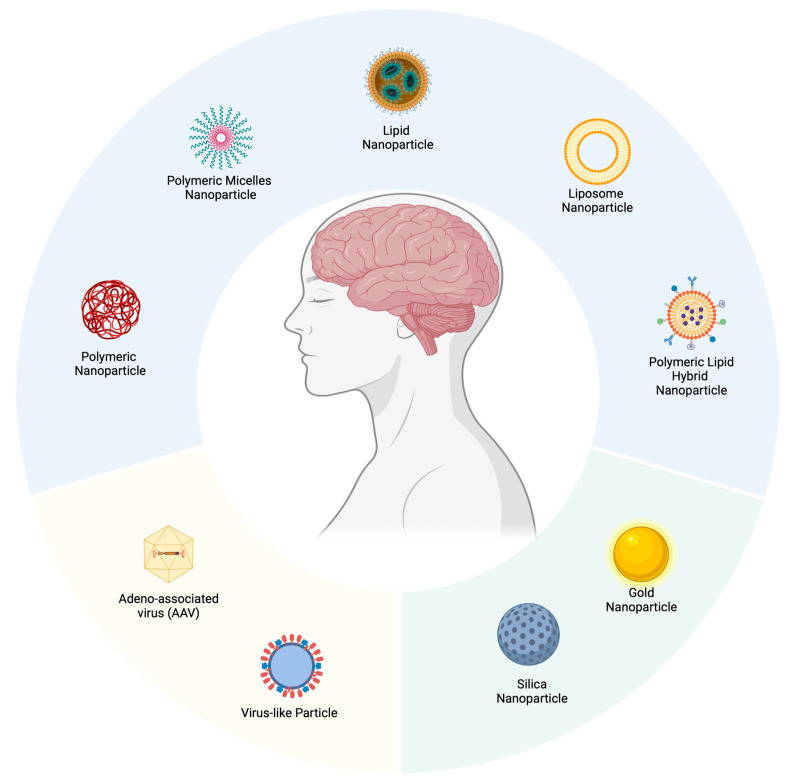
Potential nanomaterials for transporting the prime editing system to the brain. Created with BioRender.com, accessed on 7 May 2024 (Agreement Number KW26VSEVXF).

An efficient drug delivery system is urgently required to transport the PE to the brain. Diverse nanocarriers have been employed for gene delivery; however, only a few are suitable for repurposing for PE delivery. We discuss different nanocarriers (Figure 4) that could be deployed to deliver prime editing-based nanomedicine to the brain.

## 6. Viral Delivery Systems

Viral vectors have risen to prominence as eminent mechanisms for the transmission of genes, presenting an efficient mode for the insertion of genetic content into specific cells. Across the array of viral vectors dedicated to gene delivery, adenoviruses, retroviruses, lentiviruses, and adeno-associated viruses (AAVs) stand out for their comprehensive exploration and deployment in research and medical settings, as detailed by Boeck et al. [28]. Distinguished by their lack of an outer envelope and their double-stranded DNA configuration, adenoviruses demonstrate an exceptional capacity to enter cells during both the proliferation and dormancy phases, making them highly suitable for diverse applications, according to Zhi et al. [29]. However, they also prolong gene expression and integration into the genome, which could lead to off-target effects. Research by Chen et al. [32] and Levy et al. [33] sheds light on AAVs. These vectors are distinguished by their small, non-enveloped structures and single-stranded DNA, earning commendation for gene transfer applications due to their minimal immunogenic responses, absence of pathogenic effects, and versatility in infecting diverse cell types. Through strategic modifications, these viral vectors have been optimized to reduce their inherent pathogenic factors and improve their efficiency in gene delivery, notably facilitating the transportation of PE to cerebral regions [33]. To date, various AVV serotypes have been isolated, and each one has specific tropism for certain tissues or organs [121]. Among various serotypes utilized for delivering drugs to the central nervous system, AAV9 possesses a distinctive capability to cross the BBB and infect not only neurons but also non-neuronal cells [56,57,58,59,60]. While this allows for the precise targeting of neural cells, it also presents a potential risk of affecting surrounding tissues or cells, resulting in unintended off-target effects [122,123,124,125,126].

### 6.1. Advantages and Limitations of Viral Vectors

Viral vectors have demonstrated higher tropism and efficacy compared to some nonviral vectors, which has made them the prime choice for clinical application in gene therapy, as demonstrated by the current list of clinical trials deploying AAVs as the delivery system (see Table 3). Three AAV-based treatments have been authorized by the US Food and Drug Administration (FDA) to address Leber’s congenital amaurosis, spinal muscular atrophy, and hemophilia B. However, the costs for these treatments are approximately USD 850,000, USD 2,100,000, and USD 3,500,000 per treatment dose, respectively [127]. Aside from AAVs carrying a high production cost, scientists are still challenged by mitigating viral vector safety, toxicity, immunogenicity (binding of pre-existing neutralizing antibodies (NAbs)), insertional mutagenesis, allergic and inflammatory responses, and payload limitation (~4.7 kb not including the inverted terminal repeats), as well as their bioavailability, to fully cure a pathology over time without the need to administer multiple doses [128].

As indicated in the FDA’s 2021 report on Cellular, Tissue, and Gene Therapies Advisory Committee Meeting No. 70, there have been multiple cases of treatment-emergent serious adverse events in gene therapy studies with AAV8 vector-based products for an X-Linked Myotubular Myopathy [129,130]. In 2020, three patients died during a clinical trial, and there was an additional report of a fourth deceased patient in 2021 (Phase I/II ASPIRO clinical trial NCT03199469 sponsored by Astellas Gene Therapies). In a recent investigation, a 27-year-old individual with Duchenne’s muscular dystrophy (DMD) received treatment using a high dose of recombinant adeno-associated virus (rAAV) serotype 9 carrying a transgene for “dead” Staphylococcus aureus Cas9 fused to VP64 [131]. This personalized CRISPR–transactivator therapy aimed to increase cortical dystrophin expression. However, following the treatment, the patient experienced mild cardiac dysfunction and pericardial effusion, which progressed to acute respiratory distress syndrome and ultimately led to death within eight days. A postmortem analysis indicated severe diffuse alveolar damage, minimal liver transgene expression, and no presence of AAV serotype 9 antibodies or effector T-cell reactivity in the organs. These results suggest that the combination of the patient’s advanced DMD condition and the high-dose rAAV gene therapy may have triggered a fatal innate immune reaction, resulting in acute respiratory distress syndrome (ARDS).

### 6.2. Recent Advancements in Viral Vector-Based PE Delivery

PEs are approximately 6.4 kilobases (kb) in size, which exceeds the cargo limit of AAV at around 4.7 kb [128,132]. Dual-AAV vectors have emerged as a potential solution to address some limitations and challenges associated with viral vector-based gene therapy. It is also crucial to consider the efficacy of dual-AAV vectors, in which intein-split dual-AAVs with optimized packaging and delivery systems show promise in overcoming cargo size limitations and enhancing the effectiveness of viral vector-based gene therapy while raising the challenge of ensuring efficient annealing within target cells. The race to overcome these challenges is ongoing.

Recently, a team of scientists developed an AAV-PE vector with increased PE expression, prime editing guide RNA stability and the modulation of DNA repair. Their refined PE-AAV systems, especially v3em PE3-AAV, exhibited enhanced potential for CNS-specific editing, suggesting an increase in CNS editing efficiency compared to its predecessors, such as v1em PE3-AAV [133]. Using the v3em PE3-AAV9 architecture at a dose of 1 × 1011 viral genome (vg) to deliver PE3max with epegRNA for installing the Dnmt1 +1 C-to-G through i.c.v injection resulted in a successful editing rate of 42% in the bulk cortex of mice. Furthermore, their PE system was further exemplified by successfully installing a clinically relevant mutation, specifically the ApoE Christchurch (ApoE 3 R136S) variant. Specifically, Davis et al. administered 1 × 10^11^ vg (5 × 10^10^ vg per hemisphere) of v3em PE3-AAV9 via i.c.v, encapsulating the optimized epegRNA and sgRNA, to humanized ApoE3 mice. To evaluate the influence of administration timing on the transduction of non-neuronal cells—a phenomenon reported to be augmented with aging—injections were conducted at postnatal days 1 (P1) and 3 (P3). At three weeks post-administration, an assessment of prime editing efficiencies was conducted on bulk nuclei extracted from the neocortex and hippocampus, regions implicated in Alzheimer’s disease pathology. The results indicated prime editing efficiencies of 12% (with 5.0% indels) and 14% (with 3.1% indels) in the bulk neocortical and hippocampal tissues, respectively, for the P1 cohort. Comparatively, the P3 cohort demonstrated prime editing efficiencies of 8.2% (with 4.6% indels) and 7.1% (with 3.8% indels) in similar tissues. Additionally, to ascertain the installation of the ApoE3 R136S mutation in ApoE-expressing cells, total RNA was extracted from the treated and control brain tissues three weeks post-injection, followed by cDNA synthesis. A subsequent analysis revealed prime editing efficiencies of 9.4% (with 3.5% indels) in neocortical ApoE cDNA and 11% (with 2.8% indels) in hippocampal ApoE cDNA. Collectively, these findings demonstrate the potential of prime editing to induce therapeutically relevant mutations within specific CNS cell types in vivo.

In a preprint, Boeck et al. [134], reported up to 44.0% editing at the Dnmt1 locus and 28.1% editing at the Adrb1 locus in the cortex, with an average of 34.8 ± 9.8% and 14.7 ± 11.6% editing after six months, respectively, using a dual-AAV delivery system to deliver optimized intein-split PE via i.c.v. injection into the brains of mice.

Moreover, Wang et al. [135] used adenovector particles to package the optimized full-length prime editing constructs for the precise editing of the Duchenne muscular dystrophy (DMD) gene in vitro. The gene editing efficiency for point mutation reached 80% in human myoblasts and 60% in mesenchymal stem cells. They also corrected the DMD reading frames in the patient’s muscle cells and reached 14% editing efficiency. Moreover, the same system is also used to deliver the dual prime editors, which target the exon 51 deletion exon 51 deletion of *DMD*. Collectively, this system provides the feasibility of using viral vectors to deliver the PE system. However, there is a significant immunogenicity in vivo, and cytotoxicity in vitro needs to be considered, which is caused by the high load of the viral gene of AdV.

These achievements provide promising opportunities for targeted gene editing in the CNS and hold great potential for therapeutic applications in neurological disorders. Davis et al. [133], conducted a study that highlighted the successful use of prime editing in the CNS, specifically targeting a clinically relevant mutation. This groundbreaking study is still in its infancy, in the feasibility phase, and its clinical safety and efficacy are yet to be assessed.

### 6.3. Virus-like Particles

Virus-like particles (VLPs) are self-assembling nanoparticles that are composed of viral protein but lack viral genetic materials, causing harmful infections; they range in size from 20 to 200 nm [136,137]. These nanoparticles possess key advantages observed in both viral and nonviral vectors. VLPs protect encapsulated cargo molecules from degradation or undesired binding in vivo. Due to their low toxicity and biodegradability, they have been approved by the FDA for use as drug carriers [138]. Despite the natural tropism of some virus-derived VLPs for CNS cells, they can be genetically modified for targeted delivery to specific cells or organs [136,139,140].

An et al. [128] optimized VLPs to deliver a prime editing system composed of CRISPR/Cas9 protein, pegRNAs, and nicking sgRNA. They developed third-generation v3 and v3b PE-engineered virus-like particles (PE-eVLPs) with up to 170-fold higher editing efficiency in human cells compared to the first-generation v1.1 PE2-eVLPs. Next, they investigated the therapeutic potential of v3 and v3b PE-eVLPs in mediating in vivo prime editing by correcting a 4 bp substitution at the Dnmt1 locus in the mouse CNS. PE3-eVLPs were injected into C57BL/6 mice via i.c.v injection on postnatal day 0 (P0), and the brain hemispheres were collected 3 weeks after injection. Also, VSV-G-pseudotyped lentiviruses containing EGFP fused to a nuclear membrane-localized Klarsicht/ANC-1/Syne-1 homology domain were also co-injected in order to select cells that interacted with eVLPs. With the v3b PE3-eVLPs, they achieved 3.2% editing in the bulk cortex and 47% average editing among green fluorescent protein (GFP) + nuclei.

## 7. Nonviral Delivery Systems

Some nonviral nanomaterials have demonstrated significant potential as a reliable delivery system for PE, offering an efficient and potentially safe alternatives to viral vectors. These materials, sourced from nature, synthetic polymers, lipids, or inorganic materials, can be specifically engineered to address the constraints associated with viral vectors [141].

### 7.1. Cationic Polymer-Based Nanoparticles

Positively charged entities, such as cationic polymers, have long been a focus of research in the delivery of nucleic acids. Polyplexes are complexes formed between different polyelectrolytes through electrostatic condensation, and they can be readily and spontaneously created by mixing nucleic acid with a cationic polymer [142]. The first ever polymer active pharmaceutical ingredient (API)-conjugated formulation was developed by Horst Jatzkewitz in 1955 [143]. Polymeric materials play a significant role in providing an alternative gene delivery system to the viral-based approaches by overcoming some viral-based gene delivery system obstacles and limitations. Cationic polymers can be engineered to fulfill the specific design needs for delivering nucleic acids [144]. Polymeric NPs can provide high efficacy like their viral peers, however, to improve the low safety, toxicity, and immunogenicity found in cationic polymers. There are multiple approaches to classifying them (e.g., organic or inorganic, natural or synthetic, etc.), and there are even sub-categories of those. In Table 4, we provide examples of some polymeric materials that have been used as neurotherapeutic nanocarriers for CNS. These polymers have either been employed as a solo material or, in most cases, as a copolymer with conjugation to other polymeric or lipidic materials to couple their capability for optimal efficacy and specificity. We specifically focused on materials that could potentially be used to implement a prime editing system in the brain.

#### 7.1.1. Chitosan Nanoparticles

Chitosan (CS), a cationic biopolymer derived from chitin, exhibits favorable characteristics for delivering nucleic acids, including electrostatic interaction with nucleic acids, biocompatibility, and susceptibility to chemical modifications. The transfection efficiency of chitosan-based systems has been demonstrated to be influenced by the precise interplay of various CS parameters, such as the charge density or degree of deacetylation (DDA), molecular weight (Mn), the ratio of amine groups to phosphate groups (N:P), and environmental factors like pH and serum proteins [145,146,147,148,149]. As demonstrated in Table 3, this naturally occurring linear copolymer consists of 2-amino-2-deoxy-D-glucopyranose and 2-acetamido-2-deoxy-D-glucopyranose. These units are interconnected through β(1→4) glycosidic linkages [147]. Due to its advantageous properties such as non-toxicity, biodegradability, biocompatibility, sustainability, functionalization, ease of manufacturing, and cost-effectiveness, CS has gained prominence in diverse applications ranging from biological and industrial uses to innovative drug delivery systems [147]. The FDA has granted chitosan GRAS status [145], and it has been approved for a range of applications. Given CS’s affinity for nucleic acid and self-assembly, as well as its safety profile, CS-based delivery systems have been utilized as a gene delivery system to transport DNA, pDNA, and RNA entities like siRNA and mRNA, both in vitro and in vivo [143,148,149,150,151]. Self-assembly is known as polyplexation, in which cationic CS interacts electrostatically with the negatively charged phosphate backbone of nucleic acids, resulting in the creation of nanoscale polyplexes [152].

CS is commonly transported via an active endocytosis transport process, specifically through phagocytosis and pinocytosis pathways. In addition, chitosan uptake through pinocytosis can be categorized into caveolin-mediated, cadherin-mediated, and clathrin-mediated mechanisms [153,154]. CS nanoparticles (CNPs) can escape the endosome and lysosome via the proton sponge effect [147,154,155].

The adoption of CS-based nanoparticles as a safe and efficient alternative to viral delivery systems has increasingly gained traction in recent years, particularly through patient-friendly, non-invasive intranasal administration routes [156,157]. Few recent studies have used a CS-based delivery system to carry CRISPR/Cas9. For instance, Khademi et al., 2022 [158], developed a versatile delivery system composed of aptamer (Apt), CS, and hyaluronic acid (HA) for transporting plasmid CRISPR/Cas9 to suppress the *FOXM1* gene. The Apt-HA-CS-CRISPR/Cas9 construct effectively targeted and internalized into specific cancer cells, facilitating the efficient delivery of CRISPR/Cas9 to the tumor while minimizing the distribution across other organs. In vivo studies revealed a significant tumor inhibitory effect of Apt-HA-CS-CRISPR/Cas9, suggesting its potential as a precise in vivo gene editing therapeutic with minimal side effects.

To enhance the suitability of CS for use in the complex environment of human blood physiology and to address its limited solubility, CS’s surface is often coated with hydrophilic polymers like PEG or HA [150,159]. This surface modification neutralizes the particle surface charge, thereby minimizing interactions with negatively charged blood components such as proteins and red blood cells. As a result, issues related to hematotoxicity, hemolysis, and hemagglutination are mitigated [148]. Through such modifications, it becomes feasible to develop formulations that exhibit enhanced hemocompatibility, which can lead to improved pharmacokinetic profiles for CS-based polyplexes.

HA, a biocompatible glycosaminoglycan, has been demonstrated to electrostatically cover CS and enhance NP compatibility with blood [146]. Furthermore, HA can improve cellular uptake by interacting with CD44 and RHAMM receptors in several cell types, including neurons, astrocytes, microglia, and oligodendrocytes, in the brain. This makes it a desirable option for reengineering the surface of nanoparticles and developing brain-targeting formulations [160].

**Table 4:** Common polymeric materials utilized in targeting CNS.

Polymers	Advantages	Structure *	References
Chitosan (CS) Hyaluronic Acid (HA)	Smart polymer, easy to reengineer, easy synthesis, controlled and targeted drug delivery, prolonged systemic exposure, biodegradable, improved bioavailability, bio-renewable, high loading capacity, low production cost, potential for high TE, near-zero immunogenic reaction, and near-zero toxicity.	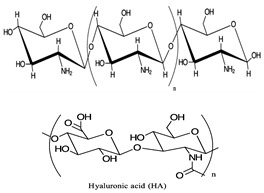	[152,153]
Polyethylene glycol (PEG) Polyethylenimine (PEI) Polylactic acid (PLA)	Easy synthesis, prolonged systemic exposure, improved bioavailability, high loading capacity, high biocompatibility profile, responsiveness to external stimuli, and high distribution in lesion tissue.	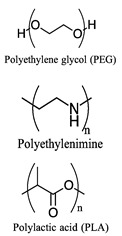	[154,155,156,157,158,159,160]

* Chemical structures created with ChemDoodle Version 12.6.0.

#### 7.1.2. Polyethylenimine Nanoparticles

The cationic synthetic polymer polyethyleneimine (PEI) has been extensively employed for nonviral in vitro and in vivo gene therapy studies, and it has an advantage over other polycations because it combines a significant nucleic acid compaction capacity with an intrinsic endosomolytic activity [161]. For instance, Sheikh et al. [162] developed polylysine-modified polyethyleneimine (PEI-PLL) for the transportation of the VEGF gene to examine its effect utilizing both in vitro and in vivo for PD. In their in vitro studies, they employed a 6-hydroxydopamine (6-OHDA)-mediated cell death model using MN9D cells transfected with either a control plasmid or a plasmid expressing VEGF. PEI-PLL-mediated *VEGF* gene transfer to MN9D cells improved cell viability, increased tyrosine hydroxylase (TH)-positive cells, and reduced apoptosis in response to 6-OHDA. Furthermore, Sheikh et al. [162] investigated the therapeutic potential of PEI-PLL-mediated VEGF gene delivery in substantia nigra pars compacta (SNPc) using the unilateral 6-OHDA medial forebrain bundle (MFB) lesion model of PD in rats. According to a behavioral study (i.e., elevated body swing test (EBST)) they conducted, *VEGF* treatment reversed the loss of motor capabilities produced by 6-OHDA. TH immunostaining revealed that *VEGF* prevented the 6-OHDA-mediated loss of DA neurons in the substantia nigra pars compacta (SNPc) and DA nerve fibers in striatum. In addition, PEI-PLL-mediated *VEGF* gene delivery inhibited apoptosis and microglial activation in their rat Parkinson’s model. Their data conclusively indicated the positive effects of PEI-PLL-mediated *VEGF* gene delivery on the dopaminergic system in both in vitro and in vivo models [162].

Polymeric materials have also demonstrated potential when formulated as conjugates in combination to serve a purpose. An example of this potential formulation is the study by Park et al. [163], in which his team formulated other delivery systems on a nonviral vector modified with rabies virus glycoprotein (RVG) and poly(mannitol-co-PEI) gene transporter (PMT). They decorated their formulation with an RVG ligand, and the PMT/siRNA complexes were delivered to the brain via an attachment to nicotinic acetylcholine receptors presented on the BBB. In their in vitro BBB model, they showed that osmotically active PMT stimulated caveolar endocytosis, improving receptor-mediated transcytosis. Using R-PEG-PMT/siBACE1 complexes, they have demonstrated that the potential of RNAi therapies for AD was proven in vitro and in vivo through the implementation of a polymeric conjugate in their formulation. Park et al.’s [163] findings indicated that R-PEG-PMT is a promising drug delivery system for brain-targeted RNAi treatments due to their decorative ligand and polymeric formulation to traverse across the BBB [163].

A salient feature of PEI nanoparticles is their ability to disrupt endosomal membranes, allowing them to bypass endosomal entrapment and release their genetic load into the cytoplasm [164]. This characteristic is crucial for PE delivery, ensuring the elements of the system reach their nuclear destination. Demonstrating their adaptability and potential, PEI nanoparticles have been successfully employed in the transport of various nucleic acid formats, like plasmid DNA, mRNA, and siRNA, underscoring their applicability in PE delivery [164]. Rohiwal et al. [165] demonstrated the direct delivery of constructs encoding Cas9 protein and gRNA using CRISPR/Cas9-PEI-MNPs, facilitating site-specific incision and the NHEJ or HDR correction of the blue fluorescent protein (BFP) and green fluorescent protein (GFP) genes. The combination of the CRISPR/Cas9-PEI-MNPs complex with an inhomogeneous magnetic field proved to be a rapid and non-toxic strategy for CRISPR/Cas9-mediated genome editing. Further research is required to assess the potential application of MNPs tailored for delivering a CRISPR/Cas9 genome editing system in in vivo conditions.

The efficiency of PEI-based magnetic nanoparticles (MNPs) for conveying plasmid DNA that encodes the CRISPR/Cas9 system into eukaryotic cells in vitro has been confirmed, emphasizing PEI nanoparticles’ utility in genomic modification undertakings [165].

However, PEI nanoparticles confront several challenges that must be surmounted to facilitate their effective utilization in delivering PE to neural tissues. A significant hindrance is the potential cytotoxic effects attributed to the high positive charge density of PEI, which could compromise cellular integrity, leading to cytotoxic outcomes [166]. To tackle this, researchers have employed strategies such as integrating PEG chains or using lower molecular weight versions of PEI, which have been shown to reduce cytotoxicity while preserving the delivery efficacy of the nanoparticles [167,168].

#### 7.1.3. Micelle Nanoparticles

Polymeric micelles (PMs) have made substantial advancements in the field of drug delivery. In terms of the ease of large-scale production, micelles are extremely attractive [169]. Their molecular structure is well-defined, and their assembly behavior is well-known [170]. This makes it easier to formulate and manufacture future treatments. The selection of the polymer type impacts the self-assembly procedure for creating PMs. They are frequently made up of a copolymer containing both hydrophilic and cationic components, enabling the incorporation of nucleic acids into PM structures [171]. PMs are micelles composed of amphiphilic macromolecules, mainly di- or tri-block copolymers made of solvophilic and solvophobic blocks [172]. One advantage of micelles is that they can be easily triggered to release therapeutic agents. Other advantages of micelles are their high biocompatibility and flexibility for design modifications [172].

Gothwal 2023 [173] utilized a CS-based polymeric micelle formulation to effectively transport pVGF to the brain and express VGF. They successfully transfected brain cells in vivo using i.n. administration in their mouse model. The researchers enhanced their formulation by incorporating oleic acid (OA), penetratin (PEN), and mannose (MAN) to construct the OA-g-CS-PEN-MAN/pVGF polyplex, which resulted in significantly higher VGF expression in the brain. This highlights the potential of this delivery system for gene therapy targeting Alzheimer’s disease.

Jiao 2018 [174] delivered the eGFP DNA plasmid to the brain via PMs. Their PMs were decorated with angiopep-2. This ligand targets the low-density lipoprotein receptor-related protein-1 (LRP1), which is overexpressed in the BBB and glioma cells. Their micelles, named ch-Kn(s-s)R8-An, were also microenvironment-responsive, utilizing the matrix metalloproteinase 2 (MMP-2)-responsive peptide as a linker to conjugate angiopep-2. MMP-2 is upregulated in the tumor microenvironment. It degrades the linker enzymatically, allows for the exposure of R8, which is a peptide that leads to internalization in the cells, and then permits penetration into the core of the tumor. Using i.v. injection, the authors delivered a dose of 50 mg of DNA per mouse, showing higher pronounced fluorescent signals than controls. Their results also showed a directional aggregation at the brain tumor site.

In 2021, Abbasi et al. [154] were the first to demonstrate genome editing in brain parenchymal cells using RNA-based delivery of CRISPR/Cas9. They covalently conjugated a polyethylene glycol (PEG) chain to the PM and constructed a PEGylated polyplex PM. The researchers successfully delivered Cas9 mRNA and sgRNA, knocking out the STOP cassette of tdTomato expression to the brain of Ai9 transgenic mice. Their results showed that with their vehicle, packaging the Cas9 mRNA and the sgRNA together induced more effective genome editing than when Cas9 mRNA and sgRNA were packaged separately. This co-encapsulation allowed for the improvement of the sgRNAs’ stability. Following intraparenchymal injection, the co-encapsulation of Cas9 mRNA and sgRNA in PMs led to efficient gene editing in a large area of the mouse brain parenchyma, including neurons, astrocytes, and microglia. The authors also demonstrated that the efficiency of editing using PMs was higher than using a non-PEGylated micelle. Therefore, they showed that by improving diffusion in brain tissues, the presence of PEG in the micelle is crucial for good gene editing efficiency. Their nanoparticles encapsulated Cas9 mRNA and sgRNA at a weight ratio of 1:1. This vehicle had a size of 64.9 nm and a PDI of 0.23.

### 7.2. Cationic Lipid-Based Nanoparticles

**Figure 5:**
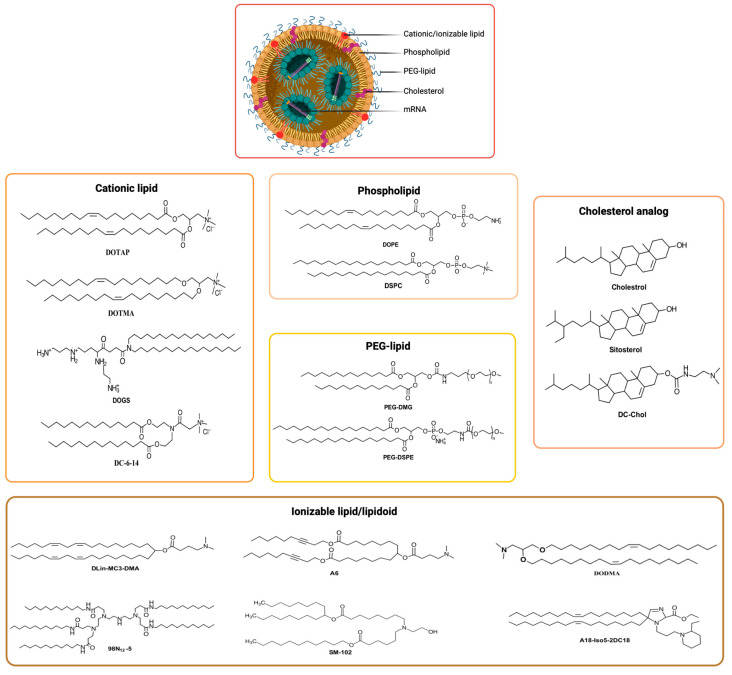
Chemical structures of typically used lipids on lipid nanoparticle (LNP) formulations. Created with BioRender.com (agreement number KH26W26572) [177,178]. Chemical structures created with ChemDoodle Version 12.6.0.

Lipids are molecules with amphiphilic properties consisting of a polar head group, a hydrophobic tail region, and a linker connecting the two domains. Lipid-based nano-delivery systems (i.e., lipid nanoparticles) usually include additional lipid components, such as phospholipids, cholesterol, or PEG (Figure 5) [175]. The primary distinctions among these nanoparticles are based on their lipid components, synthesis conditions, and the techniques employed for nucleic acid encapsulation [176].

One of the highlights of lipid nanoparticles (LNPs) (Figure 5) is their large loading capacity compared to their leading viral counterpart [179]. LNPs can encapsulate large components, such as long RNAs and large proteins [180]. This has paved the way for the delivery of gene editing therapies, such as the CRISPR technology for neurodegenerative diseases. LNPs (Figure 5) are generally synthesized from the following four classes of lipids: (i) ionizable cationic lipids, (ii) phospholipids (helper lipids), (iii) cholesterol, and (iv) PEG lipids [176,181,182,183]. Each of these lipids is important for the effectiveness of LNPs [184,185]. Ionizable cationic lipids are positively charged at an acidic pH. This feature enables the interaction with nucleic acids, which are negatively charged due to their phospholipid backbones [186], and their loading in the particle. A critical characteristic of those lipids is that they become protonated at an acidic pH. This protonation allows for the endosomal escape of the particle. In fact, when the ionizable cationic lipids become protonated, the membrane of the particle is destabilized. This lipid will then destabilize the membrane of the endosome. Consequently, the LNPs will escape from the endosomes and release their cargo into the cytosol of the cells [187]. This mechanism is similar to those of cationic polymers, such as chitosan, as explained above. Phospholipids act like « helper lipids » by improving the stability of the particle [188]. Another aspect of their usefulness is increasing delivery efficiency [189]. Cholesterol is essential in the formulation of LNPs. It increases the stability of the NPs by filling the gaps between the phospholipids [182,189]. PEG increases the circulation time of LNPs and reduces their immunogenicity [182,190,191]. The lipid composition and proportion in LNPs (Table 5) impact the specificity of the LNPs [192]. By playing on those parameters, it is possible to change the size and surface characteristics of the nanoparticles and thus influence their biodistribution. The precise mechanism that explains the relationship between the biodistribution of LNPs and their size and charge and the types of lipids used is not clearly understood. Research on this mechanism is still ongoing.

In 2018, Onpattro^®^, which is an siRNA delivered with LNP, received FDA approval [193]. In 2022, two LNP-based mRNA vaccines were authorized for emergency use to fight against the COVID-19 pandemic [194]. The application of LNPs as a drug delivery system for infectious diseases is shifting toward other diseases. Recently, Cheng et al. [195] reported a novel modifiable LNP platform called selective organ targeting (SORT) LNP, which adds a fifth lipid component to the established LNP formulation. SORT LNPs delivered different CRISPR cargoes, including mRNA, Cas9 mRNA-sgRNA, and Cas9 ribonucleoprotein (RNP) complexes, for efficient genome editing in the liver, lungs, and spleen after i.v. administration. Although this strategy is very promising in expanding the usefulness of LNPs, the possible reason why SORT LNPs preferentially accumulate in the liver is that they are easily opsonized and captured by the hepatocytes, partially due to ApoE binding to the LNPs in the blood steam [196]. In addition, selective delivery to the lungs may result from the positive surface charge of intravascular SORT LNPs [195]. Therefore, to achieve more success in other tissues, such as the brain, stealth SORT LNPs will be needed [195]. This could potentially be a promising start for the application of precision LNPs to target the brain. Han et al., 2023, for their development of a high-throughput screening platform (HTS-BBB) for the dual screening of mRNA LNP transfection of and transport across the BBB, developed strains of LNPs that reportedly crossed the BBB in their mouse model and reached the brain [197].

**Table 5:** LNP formulations delivering to the brain in vivo.

Delivered Cargo	Route	Dose	LNP Formulation	Lipids Molar Ratios	Particle Size (nm)	Reference
ASO targeting tau mRNA	i.v.	1 mg/kg	306-O12B-3	67.2 (w)	∼175	[198]
			DSPE-PEG	4 (w)		
			NT1-O14B	28.8 (w)		
(-27)GFP-Cre protein	i.v.	50 μg per injection	PBA-Q76-O16B	67.2 (w)	∼140	
			DSPE-PEG	4 (w)		
			NT1-O14B	28.8 (w)		
RNP	i.c. *	0.15 mg/kg sgRNA	5A2-SC8	21.4		[199]
			DOPE	21.4		
			Cholesterol	42.8		
			DMG-PEG	4.3		
			DOTAP	10		
DNA encoding mCherry	i.c.v. **		YSK05	70		[200]
			Cholesterol	30		
			DMG-PEG	3		

* Intracanial, ** Intracelebrovasculars.

To date, only one study has successfully packaged the PE system into lipid nanoparticles (LNPs) by delivering PE mRNA and chemical-engineered pegRNA for in vivo treatment. Chen et al. [201] achieved a 13% correction of the pcsk9 gene, which encodes proprotein convertase subtilisin/kexin type 9, an enzyme that degrades low-density lipoprotein receptors (LDLRs). The treatment was administered to immunodeficient mice using a retro-orbital intravenous injection of LNPs containing prime editing components. The authors aimed at the insertion of 4 bp (TTAC) into the pcsk9 gene to introduce a premature termination codon and thus shift the reading frame to inactivate the gene. Inactivating the PCSK9 gene is a strategy of choice in the treatment of hypercholesterolemia because it reduces blood levels of LDL-cholesterol (LDL-C) without inducing deleterious effects. In the study, scientists demonstrated that the use of LNPs to deliver the PEs increased the gene editing efficiency of the prime editing system for in vivo treatment by 2.8-fold.

### 7.3. Inorganic Nanoparticles

#### 7.3.1. Metal Nanoparticles

Owing to their unique attributes and versatility, metal nanoparticles are a subset of inorganic nanoparticles [202]. Metal NPs have been utilized as gene delivery vectors, with noble metals such as gold and silver constituting a portion of their composition [203]. These metals contain surface plasmon resonance that enables precise sensing; their surface is easily biofunctionalizable, permitting various applications such as light-triggered events. The key benefits of these systems are their simplicity to synthesize, well-defined compositions, and high-biocompatibility profiles [204].

#### 7.3.2. Gold Nanoparticles

Gold nanoparticles, also known as AuNPs, consist of gold (Au) and have a diameter that commonly falls between 1 and 100 nm [205]. AuNPs are highly stable and widely used in several applications, including theragnostic and gene therapy [205]. Shahbazi et al. [206] synthesized AuNPs using the citrate reduction method. They then developed a CRISPR nanoformulation by combining the AuNPs with guide RNA and nuclease on their surfaces. This nanoformulation could either include or exclude a single-strand DNA (ssDNA) template, which is used to facilitate homology-directed repair [206]. The result demonstrated a highly effective gene editing modification. Additionally, Shahbazi et al. [206] showcased the safe transport of complete CRISPR sequences into human blood stem and progenitor cells without toxicity.

#### 7.3.3. Silica Nanoparticles

Silicon dioxide, or silica, NPs are commonly employed to transport biologics or medications [207]. Their particle sizes, shapes, and porosities can be reengineered, and their surfaces can be optimized by coating them with cationic polymers (i.e., PEI, dendrimers, and cationic lipids). At the same time, silica NPs with pore sizes larger than 15 nm and functionalized surfaces have been considered for incorporation with positively charged primary amine groups to ensure higher encapsulation and loading efficiency, as well as higher nucleases.

Hence, it is conceivable to engineer them to produce NPs with extended circulation durations, excellent targeting features, high drug loading capacities, adequate cellular absorption profiles, and minimal toxicity. Furthermore, these systems have excellent storage stability and are inexpensive and simple to prepare in large quantities [207]. The most attractive characteristic of these systems is their ability to store and release a wide variety of drugs and to provide a large surface for storing drugs and NAs with a changing pH, allowing for the incorporation of hydrophilic and hydrophobic molecules; the latter is difficult to deliver with other systems.

Wang et al. [208] developed a water-in-oil microemulsion to create and screen a library of glutathione-responsive silica nanocapsules for the targeted delivery of biologics to the brain, such as DNA, mRNA, and Cas9 RNP. Wang et al., in their in vivo studies, showed that SNCs conjugated with glucose and rabies virus glycoprotein peptide can effectively bypass the intact BBB when systemically delivered under glycemic control, enabling the widespread delivery of various biologics, including CRISPR genome editors targeting different genes, in both Ai14 reporter mice and wild-type mice. The results demonstrated neuron editing via the systemic delivery of Cre mRNA in Ai14 mice, a reduction in amyloid precursor protein gene expression by up to 19.1%, and a reduction in tyrosine hydroxylase gene expression by up to 30.3% in wild-type mice. This adaptable SNC nanoplatform presents a promising approach for treating neurodegenerative disorders such as AD, PD, and HD.

## 8. Advanced Delivery Strategies

### 8.1. Blood–Brain Barrier Disruption Techniques: Enhancing PE Delivery

Advanced delivery strategies for PE to the brain encompass various approaches to triumph over the BBB, together with transient disruption strategies. Numerous methods have been created over time to overcome the challenges associated with BBB and transport the API to the CNS. These approaches include direct injection into the CNS (i.e., i.c.v. injection), the modification of drug molecules to improve permeability, molecular Trojan horses for receptor-mediated transcytosis, biochemical BBB disruption using substances like mannitol, nanoparticle-mediated delivery (i.e., polymeric -and-lipid nanoparticles), focused ultrasound (FUS) with microbubbles, magnetic resonance imaging (MRI) with FUS, and other techniques such as electromagnetic field modulation and vasoactive chemicals (i.e., bradykinin, histamine) [209]. These techniques can provide the benefit of permitting the delivery of PE to precise brain regions, likely decreasing systemic aspect consequences. However, they also have limitations, which include the ability for off-target complications and the need for cautious monitoring and manipulation of the disruption method to limit tissue harm [210].

### 8.2. Blood–Brain Barrier Circumvention Approaches: Alternative Routes for PE Delivery

**Figure 6:**
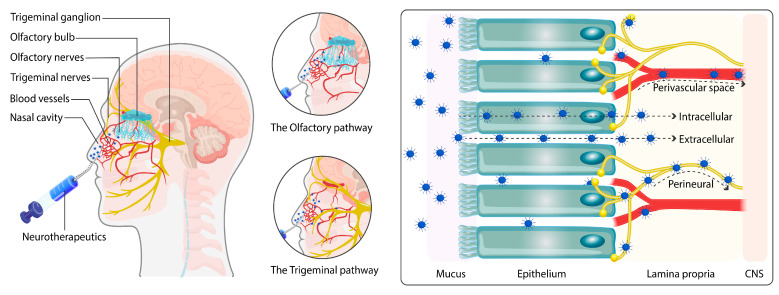
Intranasal delivery routes. OfN and TgN routes are two promising pathways for targeted local drug delivery, which have demonstrated the capability to bypass the blood–brain barrier, enabling therapeutics to traverse via perivascular, intracellular, extracellular, and perineural space from NtB [213,214,215]. Created with BioRender.com, accessed on 7 May 2024 (Agreement Number RP26VSF062).

When a neurotherapeutic drug is administered into the nasal cavity (Figure 6), at first, the mucociliary clearance in the vestibular area is the first to encounter it. The respiratory region (the most significant portion of the nasal cavity) is thus abundantly supplied with blood vessels and trigeminal nerves. In this location, medication absorption occurs in the following two ways: first, through the direct neural channel of trigeminal nerves (TgNs), and second, through the indirect route of systemic circulation [211]. The nasal cavity’s most posterior area is the olfactory region, which is related to the brain via olfactory nerves (OfNs). Along with the TgN pathway, the OfN pathway is believed to be the principal route for medication transport from the nose to the brain (NtB) [212].

The OfN route is favored simply because it can circumvent the blood barriers. Its other benefits include that it avoids first-pass metabolism and it has a non-invasive nature, quick and rapid absorption, high surface nasal area, an early initiation of the action, reduced systemic exposure, and limited adverse effects [216,217]. Drugs with a high molecular weight, such as proteins and stem cells, can be delivered through the NtB route. They can treat several disorders, such as PD, AD, epilepsy, and primary brain malignancies [218]. In addition, i.n. route treatment requires lower volumes of therapeutic drugs and can be self-administered, and doses may be adjusted compared to parenteral or oral therapies [219,220]. The efficacy of neurotherapeutic drug delivery via the i.n route has already been tested for neuropathologies.

In recent years, the NtB drug delivery pathway has gained much attention for gene therapy targeting CNS. This delivery route has been extensively studied in the management of NDs. Oxytocin and insulin are likely the only two drugs whose application for AD and PD management have been researched via intranasal drug administration [221,222]. Their applications to bring relief to certain symptoms have been studied [223].

The administration of oxytocin via i.n. and i.v. routes reveals that oxytocin administered intravenously is directly transported to the brain and that the social–cognitive benefits of i.v. oxytocin are not primarily attributable to peripheral oxytocin receptor actions. In particular, Quintana et al. revealed that despite equal peripheral oxytocin levels after i.v. and i.n. injection (delivered using a double-dummy methodology), social–cognitive and neurological effects were only detected following i.n. administration [224]. In 2023, the United States FDA approved the first over-the-counter MOR antagonist, Naloxone^®^ nasal spray, as an essential emergency treatment for reversing opioid overdose [225].

Furthermore, the NtB administration route of active pharmaceutical ingredients (APIs) for neurodegenerative diseases such as AD has been widely implemented, whereas multiple clinical trials (i.e., NCT03857321) with i.n.-administered insulin (Humulin^®^) have been conducted [226].

Through the i.n. route, Dhaliwal et al. [227] delivered mRNA to the brain via liposomes. Their particles were composed of DPPC/DOTAP/cholesterol at a molar ratio of 5/5/3. They made a liposome encapsulating GFP mRNA and another one encapsulating luc-mRNA, with particle sizes of 195 and 222 nm, PDIs of 0.19 and 0.20, **ζ**-potentials of 35.6 mV and 37 mV, and encapsulation efficiencies of 80 and 76%, respectively. They injected these particles intranasally into CD-1 mice. Liposomes containing GFP mRNA were injected at a dose of 3 mg/kg and showed a 15% higher expression compared to the control group. For luc-mRNA, when injected at 3 mg/kg, luciferase activity was significantly enhanced in the cortex region by 21-fold and 12-fold, compared with the empty liposomes and the naked luc-mRNA group.

In contrast, others reported that the nasal route has a few drawbacks, including a restricted administration volume, patient noncompliance, and a short residence duration due to rapid mucociliary clearance [215,228,229,230].

Ndeupen et al. [231], in their SARS-CoV-2 preclinical vaccine studies using mouse models, evaluated the immunological response of Acuitas’s LNPs carrying mRNA. They delivered their API through three intradermal (i.d.), intramuscular (i.m.), and i.n. deliveries for their preclinical studies. Ndeupen et al. [231], reported that LNPs induced quick and powerful inflammatory responses, characterized by significant neutrophil infiltration, the activation of multiple inflammatory pathways, and the generation of numerous inflammatory cytokines and chemokines when injected i.d. or i.m. The same amount of LNPs administered i.n. produced similar inflammatory reactions in the lungs, with a higher death rate, although the underlying mechanism is unknown [231]. Moreover, in their i.n. API dose–toxicity study, they delivered LNPs to wild-type B6 mice ranging from 2.5 mg to 10 mg/mouse and assessed their health and weight for eight days post-administration [231]. They observed a correlation between survival rates as they increased the dose. Moreover, they discovered that 80% of mice that were administered 10 mg of LNPs died within 24 h of administration. Their results showed that the 5 mg dosage killed 20% of the mice, but subjects treated with 2.5 mg survived and showed no substantial weight loss or clinical symptoms of distress [231].

Moreover, researchers must contend with possible drawbacks of this administration route, such as the limited membrane permeability of nasal epithelium and the brief residence period in the nasal cavity.

The recent trend toward deploying polymeric and lipidic materials for neurodegenerative gene therapy applications indicates their promising potential (Table 6). Many research groups have successfully used nonviral vectors in various preclinical applications for treating brain cancers and PD and cellular reprogramming for neuron replacement [106]. Table 7 presents preclinical studies aimed at drug discovery for neurodegenerative diseases using polymeric and lipidic materials.

### 8.3. Targeted Delivery Systems: Enhancing Specificity and Efficiency

Precision targeting of nanomedicine requires engineering both the drug delivery system and the PE system to enhance specificity and efficiency in delivering to the target tissues or cells. CRISPR/Cas9 technologies, such as PE, are prone to off-target effects, which can lead to unintended mutations or disruptions in the genome. PE has shown promise in addressing these limitations by providing a more precise and controlled editing platform. However, by designing formulations for precision targeting and selecting a proper delivery route, the potential risks of off-target effects can be minimized, ensuring the safe and effective delivery of PE in therapeutic applications.

#### 8.3.1. Stimuli-Responsive Material Engineering: Enhancing Specificity and Efficiency

To enhance the precision and efficacy of delivering PE to the brain, scientists have developed stimuli-responsive and targeted nanoparticle systems. Nanoparticles that respond to pH changes, such as those derived from polymers or silica, capitalize on the acidic conditions found in endosomes to initiate the release of their contents, thus improving the delivery of PE into the cytosol [208,244]. Decker et al. [245] reported a decrease in the pH in the brains of C57BL/6 mice with age. Moreover, in their research, they discovered that postmortem brain and CSF pH are even lower in cases of AD compared to those unaffected subjects. Decker et al.’s [245] in vivo experiments demonstrated that infusing low-pH CSF led to an increase in amyloid-beta (Aβ) plaque load in APP-PS1 mice. They also observed that mild acidosis reduced the release of tumor necrosis factor-alpha induced by Aβ 42 in microglia, as well as their ability to uptake this peptide. Brain acidosis is linked to aging and may impact pathological processes like Aβ aggregation or inflammation in AD [245]. pH-responsive nanomaterials provide new opportunities for targeted drug delivery systems to better exploit the changes in physiology seen in affected patients.

Furthermore, nanoparticles that are redox-responsive, activated by the elevated levels of glutathione in the brain, have been explored for their ability to ensure a controlled release and heightened bioavailability of PE [246]. Nanoparticles equipped with ligands, including antibodies, peptides, and aptamers that bind specifically to receptors on brain cells, have been proven to bolster the targeted absorption and accumulation of PE in the brain [244,247]. Additionally, external stimuli such as magnetic fields or light can be employed to navigate and trigger the discharge of PEs from nanoparticle carriers, offering precise control over gene editing activities in the brain [248].

#### 8.3.2. Precision Uptake Enhancement by Ligands: Enhancing Specificity and Efficiency

The active targeting of drug-loaded nanoparticles augments the effects of passive targeting, enabling nanoparticles to be guided to the appropriate location and, subsequently, transporting and delivering medications to the site of action, the brain. The high surface-to-volume ratio of these nanosystems enables the nanoparticles to be highly chemically reactive, allowing for surface modification with compounds that may be recognized by receptors/transporters overexpressed in the BBB and cell-specific receptors in brain tissue [249,250]. Moreover, as mentioned above, adding ligands for receptors seems to be the most efficient method for active targeting, given the high ligand–receptor specificity [251]. Active targeting can be carried out in a variety of ways. To actively target only tissue affected in the brain, it is necessary to understand the type of cell receptors the medicine will target [252,253,254]. There are essentially three ways to accomplish this, as follows: (i) adsorptive-mediated transcytosis, (ii) transporter-mediated transcytosis, and (iii) receptor-mediated transcytosis [253,254]. Once in the brain, the nanocarrier must be able to reach its intended target, brain tissue or cells such as neurons, glial cells, or the amyloid fibrils linked to several neurological disorders.

One of the most remarkable contributions to CNS drug delivery was introduced by Kataoka et al., who addressed the issue of off-targeting associated with systemically injected nanocarriers, which may also accumulate in the endothelia of peripheral organs, in addition to their main encephalic targeted sites. This team brilliantly exploited the lower endocytic rate of the cephalic endothelium to increase preferential retention of protein-binding ligands (i.e., labeled endothelium) on the surface of the brain endothelium relative to the peripheral endothelium. Consequently, nanoparticles capable of successfully binding to the ligands are specifically targeted to the brain endothelium with low accumulation in peripheral organs [255].

Scientists can use cell-specific ligands (Table 8) to enable the nanoparticle to attach selectively to the cell that possesses the corresponding receptor. Using transferrin as the cell-specific ligand, this method of active targeting was discovered to be effective [252]. The global peptide therapeutics market was worth USD 25 billion in 2018 and is expected to grow to USD 49.5 billion by 2027 [256]. The primary benefits of neurotherapeutic peptides are their high potency and selectivity, limited number of side effects, specificity for their target receptors, limited drug–drug interactions, low immunogenicity, and nuclear entry [257,258].

To further reengineer the polymeric and lipidic nano-delivery system for neurotherapeutic gene therapy, ligands such as cell-penetrating peptides (CPPs) or other ligand classes can be deployed to enhance the formulation surface and further increase their transfection efficacy and CNS target specificity. Researchers established that CPPs, which are made of 5–30 a.a., enhance cellular penetration and uptake in brain cells for drug delivery systems [259]. For instance, the CPPs and TAT peptides can deliver proteins, DNA, and nanoparticles (NPs) into the nucleus [260]. TAT (YGRKKRRQRRR), which contains six arginine and two lysine residues and therefore possesses a high net positive charge at physiological pH levels, has been shown to increase brain cell expression and uptake efficacy for neurodegenerative therapeutic application drug targeted delivery systems [261,262]. Due to the TAT structure and method for synthetization, it can conjugate covalently or non-covalently with cationic polymers and large-sized proteins. The application of the CS-PEG-TAT formulation in siRNA delivery was successfully tested as a potential intracellular targeted drug delivery system for neurodegenerative diseases in in vivo models; furthermore, Malhotra et al. concluded that they delivered 0.5 mg/kg of siRNA four hours post i.n. delivery to the hippocampus, thalamus, hypothalamus, and Purkinje cells in the mouse brain [159,263].

In another study, the CPPs and TAT peptides were used to study intranasal siRNA administration with polyethylene glycol-polycaprolactone (PEG-PCL) micelles. The brain distribution of FAM-siRNA was much more significant after i.n. administration compared to i.v. administration, and the coupling of TAT to MPEG-PCL enhanced the transmucosal effectiveness of the gene carrier [264]. In another investigation of i.n. administration, nanoemulsion was utilized as a mucoadhesive to lengthen the period that nanoparticles remained intact with nasal mucosa, which increased siRNA endocytosis. In a model of neuroinflammation, siRNA was found in the brain up to 24 h after the nasal injection of nanoemulsions [265]. Other studies have demonstrated the potential of olfactory i.v. in the in vivo delivery of siRNA encapsulated and decorated with CS-PEG-TAT nanocarrier into the brain of their animal model [232].

Topal et al. [266] conducted research to develop a nanoscale drug delivery system for a more efficient transfer of donepezil, an anticholinergic medication used in the treatment of AD, across the BBB. Apolipoprotein E (ApoE), a ligand of BBB receptors, was used to target RhB-labeled solid lipid nanoparticles carrying Aricept ODT^®^ (Donepezil). Their cellular uptake studies of the SLN cargo in rat and human BBB models and SH-SY5Y neuronal cells revealed an increase in RhB cargo in all tested cell types in the presence of ApoE targeting ligand on the surface of their formulation. For instance, in primary rat brain endothelial cells, the uptake of RhB-labeled donepezil cargo packaged in ApoE-targeted NPs was greater than fourfold (463%) higher than the uptake of cargo enclosed in non-targeted particles after 2 h of incubation [266].

Moreover, when targeting the brain with LNPs, two significant problems need to be addressed. Primarily, when these particles are injected i.v., they tend to be trapped by the liver and lead to what is known as hepato-cytotoxicity. The discontinuous endothelium explains this phenomenon in one part in the liver and in another part in the biological effect of apolipoprotein E (ApoE) [267,268]. When LNPs are in blood circulation, ApoE can form a corona around them. LNPs are then directed toward the liver because ApoE binds specifically to hepatocyte receptors. The second complication is that the LNPs need to pass through the BBB.

To face the latter problem, Ma et al. [198] developed a class of neurotransmitter-derived lipidoids (NT-lipidoids). When conjugated to LNPs, initially unable to permeate the BBB, these NT-lipidoids enable passage through the BBB. For instance, with the NT-lipidoid NT1-O14B, Ma et al. [198] have successfully delivered antisense oligonucleotides (ASOs) against tau and the genome editing fusion protein (−27)GFP-Cre recombinase to the mouse brain via i.v. injection. To deliver ASOs against tau, their LNP was composed of 306-O12B-3/DSPE-PEG/NT1-O14B at a ratio of 67.2/4/28.8 (*w*/*w*), with a total lipid/ASO ratio of 15:1. With this formulation, Ma et al. obtained an approximately 50% reduction in tau mRNA and an approximately 30% reduction in tau protein. To deliver the genome editing fusion protein (−27)GFP-Cre recombinase, the researchers injected the following formulations into Ai14 mice: PBA-Q76-O16B/DSPE-PEG/NT1-O14B with a ratio of 67.2/4/28.8 (*w*/*w*). Strong tdTomato signals were observed in multiple brain regions, including the cerebral cortex, hippocampus, and cerebellum. As these particles seem to be very effective, it would be relevant to test their specificity to verify that they do not travel to the liver or other organs.

**Table 8:** List of ligands deployed in CNS targeting.

Ligand	Receptor(s)/Target(s)	Refs.
β55 (aptamer)	Aβ40 fibril	[269]
c-abp2, n-abp4	Aβ42 oligomer	[270]
N2, E2 (aptamer)	Aβ40 monomer	[271]
E22P-AbD43 (aptamer)	Aβ42 dimer	[272]
Selegiline	Amyloid-beta peptide	[273]
TAT (CPP)	Cell membrane (translocation)	[159,232,263]
Curcumin	Amyloid-beta peptide	[274,275,276]
Sialic acid	Cell membrane	[277,278]
Solanum tuberosum lectin	N-Acetylglucosamine	[279,280]
Odorranalectin	L-fucose	[281,282,283]
Transferrin (Tf)	Transferring receptor (TfR)	[284,285]
Lactoferrin (Lf)	Lactoferrin receptor (LfR)	[285]
g7 Peptide	BBB	[286]
Opioid peptides	BBB	[287]
Syn-B	-	[288]
CDX peptides	Nicotine acetylcholine receptors (nAChR)	[289]
Angiopep-2	LRP	[290,291]
TGN peptide	BBB	[292]
ApoE	LDL receptor (BBB)	[293,294,295]
IGF1R5	IGF1R	[296]
OX26 R17217	Transferrin receptor (TfR)	[297,298,299]
Anti CD44 mAB	Glial cells	[300,301]
Anti NCAM1 mAB	Neurons	[302]
FD7	E-cadherin, BBB	[303]
CCD	BBB	[303]

#### 8.3.3. Enhancing Prime Editing: Increasing Specificity and Reducing Off-Target

Scientists have refined methodologies to bolster the effectiveness and precision of prime editing systems, ensuring superior delivery to designated cells. Antoniou et al. [304] have illustrated advancements in nuclease-driven prime editing through the modulation of DNA repair mechanisms and the customization of pegRNAs, resulting in the enhanced accuracy and efficacy of the editing process. Choi et al. [305] introduced the concept of paired PE, which enables precise genomic deletions by using two pegRNAs targeting opposite DNA strands, expanding the versatility of this technology. Dirkx et al. [306], achieved increased PE gene modification rates in KCNQ2 and SCN1A genes using single-nicking all-in-one plasmids, simplifying the delivery of PE components. Huang et al. [307] developed a refined uni-vector prime editing system that improves editing outcomes in mammalian cells by incorporating all the necessary components into a single vector, streamlining the delivery process. Petri et al. [308] demonstrated the feasibility of CRISPR prime editing using RNP complexes in zebrafish and primary human cells, providing an alternative delivery method that avoids the challenges associated with vector-based delivery. Finally, Qi et al. [309] developed an optimized prime editing system for the efficient modification of the pig genome, highlighting the potential of prime editing for agricultural and biomedical applications. Qi et al. [309] made changes to pegRNA by increasing the length of the duplex and altering a thymine base within a sequence of consecutive thymine bases to cytosine. This significantly boosted prime editing efficiency by enhancing both the expression of pegRNA and targeted cleavage. Next, they focused on SAMHD1, an enzyme that hinders retroviral reverse transcription. Qi et al. observed that treatment with its inhibitor, cephalosporin C zinc salt, led to a substantial increase in prime editing efficiency, possibly by improving the reverse transcription process carried out by Moloney murine leukemia virus reverse transcriptase within the prime editing system. Furthermore, treatment with various histone deacetylase inhibitors notably enhanced prime editing efficiency. Among these HDACis, panobinostat was particularly effective due to its significant boost in transgene expression, leading to an up to 122-fold improvement in efficiency on average (sevenfold). Additionally, combining all three strategies resulted in further enhancement of prime editing efficiency in porcine embryonic fibroblasts.

## 9. Clinical Implications and Future Perspectives

### 9.1. Potential Therapeutic Applications of Prime Editing in Neurological Disorders

PE holds great potential for the treatment of numerous neurological issues because it allows for the right correction of disease-causing mutations and the modulation of gene expression [72]. In AD, PE will be used to correct mutations in genes, along with *APP*, *PSEN1*, and *PSEN2*, which are associated with familial AD [10,310]. Similarly, in pathologies such as PD, prime editing should be used to target mutations in genes like SNCA, LRRK2, and PRKN, offering a potential disease-enhancing approach [311]. Although there are some point mutations associated with ASD, this genetic disorder is a complex neurodevelopmental condition that frequently involves complicated genetic alterations. PE may also offer versatility in modulating not only point mutations but also ASDs with more than one gene simultaneously. Moreover, prime editing has the capability of dealing with a huge variety of different neurological diseases, including Huntington’s disease, amyotrophic lateral sclerosis, and uncommon genetic problems, by correcting the underlying genetic defects [312,313].

### 9.2. Challenges and Considerations for Clinical Translation

Drug delivery systems play a critical role in the overall effectiveness of the prime editing system. As previously discussed, given the potential hurdles associated with AAVs in human studies, innovative hybrid vectors could be created through various engineering methods to enhance their delivery efficiency, decrease immunogenicity, and improve cell or tissue specificity while minimizing off-target effects [314,315]. VLPs have also shown limitations as a potential drug delivery system, such as challenges in targeting specific cells, despite achieving significant gene editing within those cells, and their limitations must be addressed prior to their clinical applications. As mentioned previously, researchers have encountered difficulties in targeting cells when using VLPs, despite achieving significant gene editing within the targeted cells. An et al. [128], after the optimization of a PE3-eVLPs system and through i.c.v. injection, they observed only 3.2% editing in the bulk cortex. Although researchers paved the way for in vivo delivery of PE via VLPs, VLPs still require more optimization to be able to deliver PE packages to the brain.

Nonviral delivery technologies can be used to deliver PEs with precision to targeted cells and tissues while minimizing immune responses. In this endeavor, engineers, formulation chemists, and pharmacists face the significant task of designing a delivery system that can effectively co-encapsulate and transport all PE components, including CRISPR/Cas9, epegRNA, and sgRNA, each with distinct physiochemical properties. 

CRISPR/Cas9 technologies are gradually being translated into clinical settings. CASGEVY^®^, the first FDA-approved gene therapy utilizing CRISPR/Cas9 technology, is a procedure that involves modifying the patient’s own hematopoietic stem and progenitor cells ex vivo [316]. Soon, we are about to witness the initial implementation of prime editing technology in clinical trials [16]. Among many key elements for its clinical translation, immunogenicity poses an additional significant hurdle since elements of the prime editing apparatus, like the Cas9 enzyme and the pegRNA, could trigger immune reactions, potentially reducing the treatment’s effectiveness and safety [317,318]. The risk of off-target alterations, leading to accidental genetic changes, requires thorough evaluation and mitigation to affirm the precision and safety of treatments based on prime editing, particularly in clinical gene editing applications [319,320]. To enable the widespread use of prime editing technology, challenges of scalability, production, and cost need to be addressed, such as ensuring the consistent and high-quality production of prime editing components.

Regulatory challenges and concerns about safety stand as significant barriers due to the unclear short- to long-term impact of prime editing on human health and the possibility of off-target genetic edits that have yet to be comprehensively understood [317,321,322]. For instance, the biological forms of PE (such as RNP, DNA, and mRNA) should be taken into consideration. With most health and drug administration authorities approving SARS-COVID mRNA-based vaccines, it seems that using PE in the mRNA form could potentially encounter fewer bioethical regulatory limitations compared to other forms of PE.

### 9.3. Future Directions and Research Opportunities

The field of medicine is based on the principle of providing safe, timely, effective, efficient, equitable, and patient-centered care to the public [323]. Both prime editing systems and the drug delivery systems transporting the PE should guarantee each of these aspects to the public. The current high cost of gene therapies raises serious concerns about their affordability [324] and undermines the principle of medicine in providing equitable care to the public. Biopharmaceutical firms need to adopt a more streamlined model for their development and production processes to effectively control the end-product cost [325]. Affordability is a crucial factor to consider during the design conception of any potential therapeutics. Examining a single factor, such as the material selection (i.e., bio-renewable or synthetic), for prime editing delivery systems can potentially reduce costs [326].

With the ongoing advancement of prime editing, a plethora of future avenues and research potentials has been identified, aimed at refining the technology’s accuracy, effectiveness, and suitability for clinical use. Current endeavors are directed toward boosting the selectivity and performance of PE via the fine-tuning of protein structures and strategic redesigning of pegRNAs [327,328]. Exploring cutting-edge nanomaterials and innovative delivery systems, notably, versatile and adaptive nanoparticles, represents a vital strategy to navigate the obstacles inherent in transporting PE into the brain [208,329]. Designing precision drug delivery systems to target specific types of tissues, neuronal cells, and brain regions continues to present a substantial challenge that needs to be overcome in order to minimize unintended off-target effects.

Employing machine learning and computational power to determine the most effective pegRNA and PE could enhance the overall therapeutic impact and offer combined benefits in treating neurological conditions [330]. The deployment of computational power in machine learning and neural networks will expand, effectively enhancing the synthesis of nanomaterials. This could lead to the extraction of essential insights into the correlation between chemical composition, allowing for improved predictions of the performance and behavior of nanomaterials while maintaining the scalability of drug delivery systems [331,332,333,334].

The rise of personalized medicine and targeted gene modification, fueled by a deeper grasp of individual genetic discrepancies and disease pathways, promises to usher in bespoke prime editing treatments targeting neuropathologies in a more patient-centric approach [3,335].

## 10. Conclusions

Prime editing has surfaced as a revolutionary advancement with substantial promise for addressing neurological conditions, particularly those affected by a point mutation. By enabling precise and versatile gene editing without the need for DBS or donor DNA, prime editing offers a promising approach to correct disease-causing mutations and modulate gene expression in the brain. However, the successful clinical translation of prime editing-based therapies for neurological disorders is hinged on the development of safe, efficient, and targeted delivery systems that can overcome the unique challenges posed by the BBB and the complex cellular environment of the brain. Numerous materials presented throughout this article demonstrate the potential for meeting the challenges of the targeted delivery of PE to the brain. Among them, polymer- and lipid-based nanoparticles have shown promise, but much work remains to be carried out. Moreover, various approaches have been demonstrated for overcoming the challenges associated with the BBB. Some of these are non-invasive and could translate into mainstream clinical applications. Through interdisciplinary collaboration and the integration of advanced technologies, clinical researchers are making significant strides in developing innovative delivery strategies and optimizing the prime editing system for neurotherapeutic applications. As the field continues to evolve, it is anticipated that prime editing will play a pivotal role in revolutionizing the treatment landscape for neurological disorders, offering hope for patients and families affected by these devastating conditions.

## Figures and Tables

**Table 3:** Clinical trials deploying AAVs in gene therapy for neurological diseases.

NCT Number	Study Status	Conditions	Interventions	Sponsor	Phases
NCT05040217	RECRUITING	Alzheimer’s Disease|Mild Cognitive Impairment	GENETIC, BIOLOGICAL: AAV2-BDNF	Mark Tuszynski, University of California, San Diego, CA, USA	PHASE1
NCT03562494	ACTIVE, NOT RECRUITING	Parkinson’s Disease	BIOLOGICAL: VY-AADC02|OTHER: Sham (Placebo) Surgery	Neurocrine Biosciences, Irvine, CA, USA	PHASE1
NCT05541627	ACTIVE, NOT RECRUITING	Huntington’s Disease	GENETIC: AB-1001 Gene Therapy	Brainvectis, a subsidiary of Asklepios BioPharmaceutical, Inc. (AskBio), Paris, France	PHASE1|PHASE2
NCT01161576	COMPLETED	Batten Disease|Late-Infantile Neuronal Ceroid Lipofuscinosis	BIOLOGICAL: AAVrh.10CUhCLN2 vector 9.0 × 10^11^ genome copies|BIOLOGICAL: AAVrh.10CUhCLN2 vector 2.85 × 10^11^ genome copies	Weill Medical College of Cornell University, New York, NY, USA	PHASE1
NCT05603312	ACTIVE, NOT RECRUITING	Parkinson’s Disease	GENETIC: AAV-GAD Dose 1|GENETIC: AAV-GAD Dose 2|PROCEDURE: Sham Surgery	MeiraGTx, LLC, New York, NY, USA	PHASE1|PHASE2
NCT04909346	TERMINATED	Ornithine Transcarbamylase Deficiency|Wilson Disease|Glycogen Storage Disease Type IA		Ultragenyx Pharmaceutical Inc., Akron, OH, USA	
NCT04167540	ACTIVE, NOT RECRUITING	Parkinson’s Disease	BIOLOGICAL: AAV2-GDNF	Brain Neurotherapy Bio, Inc., Irvine, CA, USA	PHASE1
NCT02053064	COMPLETED	Mucopolysaccharidosis Type III A|Sanfilippo Disease Type A	GENETIC: SAF-301	LYSOGENE, Le Kremlin-Bicêtre, France	PHASE1|PHASE2
NCT00643890	TERMINATED	Parkinson’s Disease	GENETIC: Bilateral surgical infusion of AAV-GAD into the subthalamic nucleus	Neurologix, Inc., Fort Lee, NJ, USA	PHASE2
NCT00195143	COMPLETED	Parkinson’s Disease	GENETIC: Surgical infusion of AAV-GAD into the subthalamic nucleus	Neurologix, Inc., Fort Lee, NJ, USA	PHASE1
NCT01301573	TERMINATED	Parkinson’s Disease	BIOLOGICAL: rAAV-GAD	Neurologix, Inc., Fort Lee, NJ, USA	
NCT00087789	COMPLETED	Alzheimer’s Disease	GENETIC: CERE-110: Adeno-Associated Virus Delivery of NGF	Sangamo Therapeutics , San Diego, CA, USA	PHASE1
NCT03505099	COMPLETED	Spinal Muscular Atrophy	BIOLOGICAL: onasemnogene abeparvovec-xioi	Novartis Gene Therapies, Bannockburn, IL, USA	PHASE3
NCT03733496	COMPLETED	Parkinson’s Disease		Neurocrine Biosciences, San Francisco, CA, USA	
NCT00229736	COMPLETED	Parkinson’s Disease	GENETIC: AAV-hAADC-2|GENETIC: AAV-hAADC-2	Genzyme, a Sanofi Company, San Francisco, CA, USA	PHASE1
NCT03634007	ACTIVE, NOT RECRUITING	Alzheimer’s Disease|Early Onset Alzheimer’s Disease	BIOLOGICAL: LX1001	Lexeo Therapeutics, New York, NY, USA	PHASE1|PHASE2
NCT04833907	ACTIVE, NOT RECRUITING	Canavan Disease	DRUG: rAAV-Olig001-ASPA|DRUG: Levetiracetam|DRUG: Prednisone	Myrtelle Inc., Dayton, OH, USA	PHASE1|PHASE2
NCT03306277	COMPLETED	SMA-Spinal Muscular Atrophy|Gene Therapy	BIOLOGICAL: Onasemnogene Abeparvovec-xioi	Novartis Gene Therapies, Bannockburn, IL, USA	PHASE3
NCT02418598	TERMINATED	Parkinson’s Disease	GENETIC: Cohort1|GENETIC: Cohort2	Jichi Medical University, Tochigi, Japan	PHASE1|PHASE2
NCT04884815	ACTIVE_NOT_RECRUITING	Wilson Disease	GENETIC: UX701|OTHER: Placebo	Ultragenyx Pharmaceutical Inc., Novato, CA, USA	PHASE1|PHASE2
NCT05740761	RECRUITING	Rett Syndrome	OTHER: CRISPR/Cas9-based gene editing combined with AAV-based gene editing in vitro	University of Siena, Siena, Italy	
NCT04998396	RECRUITING	Canavan Disease	BIOLOGICAL: AAV9 BBP-812	Aspa Therapeutics, Okland, CA, USA	PHASE1|PHASE2

**Table 6:** Selected in vivo cases of polymer- and lipid-based NtB drug delivery systems for neurodegenerative diseases.

Drug	Pathologies	Nanocarrier	Particle Size (nm)	Zeta Potential (mV)	Benefits	Reference
Donepezil	AD	CS Nanosuspension	100–200 nm	-	Increased efficiency, enhanced API retention	[232]
Estradiol	AD	CNPs	269.3 ± 31.6 nm	24.8	High brain uptake and enhanced API retention	[233]
Rivastigmine	AD	CNPs	163.7 ± 7.6 nm	38.40 ± 2.85	High brain uptake and enhanced bioavailability	[234]
Levodopa	PD	CNPs	164.5 ± 3.4 nm	19.0	Improved uptake, avoid API degradation in peripheral circulation, enhanced residence	[235]
Ropinirole HCl	PD	LNP	98.43 ± 3.3 nm	29.91 ± 2.14	Enhanced stability, reduced dosing frequency	[236]
*Ropinirole HCl*	PD	PLN *	66.22 ± 6.2 nm	28.19 ± 3.02	Improved stability, reduced dose, and dosing frequency	[236]

* Polymeric lipid hybrid nanoparticle.

**Table 7:** Various polymer- and lipid-based delivery systems and their targeted genes for neurodegenerative gene therapy.

Material	Pathology	Target Gene	Delivery System	Type of Study	Ref.
Polymer-based vectors	PD	VEGF	PEI-PLL-mediated VEGF gene delivery	Preclinical (6-OHDA VEGF lesioned rat model)	[162]
Polymer-based vectors	PD	hGDNF	Lactoferrin-modified PAMAM dendrimer mediated GDNF	Preclinical (Rotenone- hGDNF lesioned PD rat model)	[237]
Polymer-based vectors	AD	Bace1	Rabies virus glycoprotein-modified poly(mannitol-co-PEI) gene transporter-mediated Bace1 siRNA delivery	Preclinical BALB/c mice	[163]
Lipid-based vectors	AD	BDNF	Liposomal nanoparticle-mediated BDNF gene delivery	Preclinical (APP/PS1 transgenic mice)	[238]
Lipid-based vectors	AD	APOE2	Transferrin-Penetratin-modified liposomes for delivery of ApoE2	Preclinical (C57BL/6 APOE2 mice)	[239]
Nanoparticle-based vectors	PD	SNCA	Au NP-mediated silencing of SNCA expression (using RNAi technology)	Preclinical (MPTP injected mice)	[240]
Nanoparticle-based vectors	AD	Bace1	R7L10 peptide (nanocomplex)-mediated Cas9 RNP delivery targeting Bace1 (CRISPR gene editing)	Preclinical (5XFAD transgenic mice)	[241]
Nanoparticle-based vectors	PD	SNCA	Superparamagnetic nanoparticle (Fe3O4 nanoparticle)-mediated delivery of shRNA for SNCA	Preclinical (MPTP injected mice)	[242]
Nanoparticle-based vectors	Fragile X Syndrome	Grm5	CRISPR-Au-mediated delivery of Cas9 RNP to knockout Grm5	Preclinical (Fmr1 knockout mice)	[243]

## Data Availability

Not applicable.
